# Differential expression of filamin A and its clinical significance in breast cancer

**DOI:** 10.3892/ol.2013.1454

**Published:** 2013-07-09

**Authors:** HUI-MIN TIAN, XIU-HUA LIU, WEI HAN, LING-LING ZHAO, BO YUAN, CHANG-JI YUAN

**Affiliations:** 1 Cancer Center, The First Hospital of Jilin University, Changchun, Jilin 130021, P.R. China; 2 Department of Anesthesia, The First Hospital of Jilin University, Changchun, Jilin 130021, P.R. China; 3 Norman Bethune College of Medicine, Jilin University, Changchun, Jilin 130021, P.R. China

**Keywords:** breast cancer, filamin A, immunohistochemistry, reverse transcription-polymerase chain reaction

## Abstract

Changes in filamin A (FLNa) expression contribute to the development and progression of numerous malignancies. However, *in vitro* studies of breast cancer have shown conflicting results. Thus, the present study aimed to detect the expression of FLNa in breast cancer tissue samples and the association with clinicopathological data, in order to provide insightful *ex vivo* data. A total of 96 breast cancer and distant normal breast tissues and 20 benign tumor tissue specimens were subjected to immunohistochemistry or reverse transcription polymerase chain reaction (RT-PCR) analysis of FLNa expression. Clinicopathological data were collected to analyze the association with FLNa expression. The FLNa protein was overexpressed in breast cancer tissues compared with distant normal mammary gland and benign breast tissues. The FLNa protein was expressed in 63.5% of breast cancer, with positive rates of 36, 66.7 and 84.6%, respectively, in stage I, II and III breast cancer patients (P<0.05). Overexpression of the FLNa protein was associated with advanced stage, lymph node metastasis, vascular or neural invasion, menstruation state and other risk stratifications for breast cancer. The overexpression of FLNa in breast cancer was validated by RT-PCR, indicating transcriptional regulation of FLNa overexpression in breast cancer. FLNa mRNA and protein were overexpressed in breast cancer tissues, which was associated with advanced stage, lymph node metastasis and vascular or neural invasion of breast cancer, suggesting that FLNa contributes to breast cancer development and progression.

## Introduction

Breast cancer is the most significant worldwide health problem in women >35–40 years of age. Additionally, breast cancer accounts for ~1.35 million new cases and >450,000 cancer-related mortalities annually worldwide (1). Despite improved early detection, treatment options and survival, the morbidity and mortality continue to increase and numerous patients with invasive breast cancer develop metastatic diseases that eventually lead to the patient succumbing to their condition. Thus, there is an urgent requirement to search for and identify novel biomarkers to predict tumor recurrence and metastasis and to develop more novel treatment strategies to effectively control aggressive breast cancers.

To this end, the present study focused on the identification of biomarkers for breast cancer. One possible biomarker of breast cancer is filamin A (FLNa), which is a cytoskeletal protein with a molecular weight of 280 kDa (2,3) that crosslinks actin filaments into orthogonal networks. FLNa also interacts with >45 proteins and serves as the scaffold in various signaling networks (4,5). The FLNa protein, containing an integrin-β binding domain and an RAC1 binding domain, is localized in the edge of the cytoplasm, is able to cross the membrane and may even appear in the nuclei (6,7). The actin cytoskeleton is central to numerous cell functions, including the maintenance of cell shape, cell division, adhesion, motility, signal transduction and protein sorting. The alteration of FLNa expression may contribute to cancer development and progression (4,5,8,9), and previous studies have identified FLNa overexpression in a number of malignancies (10–12). For example, in lung cancer, FLNa overexpression was shown to be associated with tumor metastasis (11), and in melanoma, FLNa-positive cells had higher migration and invasion abilities compared with FLNa-negative tumor cells (12). In breast cancer, it was reported that cyclin D1 interacted with the FLNa protein to affect the migration and invasion potential of breast cancer cells (13). However, another study showed that FLNa was able to regulate focal adhesion disassembly and suppress breast cancer cell migration and invasion (14). The reason for this discrepancy or conflict in the data is unclear. To date, there have been no studies reporting FLNa expression in *ex vivo* breast cancer tissue specimens; thus, the present study was proposed in order to detect FLNa expression in breast cancer tissue samples and to identify any associations between FLNa expression and clinicopathological data. The present results may provide useful information with regard to the function of FLNa in breast cancer and the potential of FLNa as a biomarker to predict breast cancer progression.

## Materials and methods

### Tumor tissue samples

Tissue samples were recruited from 96 consecutive primary breast cancer patients who underwent surgical resection between January 2008 and January 2010 at the Department of Breast Surgery, First Hospital of Jilin University (Changchun, Jilin, China). None of these patients received chemotherapy, radiotherapy or immunotherapy prior to surgery. All the hematoxylin and eosin-stained tissue sections were re-evaluated and confirmed by two pathologists according to the World Health Organization classifications (NCCN Breast Cancer Guidelines, version 1, 2013). Of the 96 samples, 82 cases were classified as invasive breast cancer and 14 as non-invasive cancer. Paraffinized and snap-frozen tissues of distant normal breast tissue and 20 benign tumors were also included in the study as controls. Normal skin tissues were obtained from the healthy skin of the chest area of female patients who underwent benign tumor resections and were used as a positive control for FLNa expression. In addition, 30 cases of breast cancer and distant non-tumor specimens were snap-frozen in liquid nitrogen and stored at −80°C after resection for reverse transcription PCR (RT-PCR) analysis. The institutional review board of Jilin University approved the study and each patient signed a consent form agreeing to their participation in the study.

### Immunohistochemical staining

The formalin-fixed and paraffin-embedded tissue sections were prepared for immunohistochemical analysis of FLNa protein expression. Briefly, archival paraffin blocks were retrieved and 3-μm thick tissue sections were prepared. For immunohistochemistry, the sections were deparaffinized in xylene and rehydrated in a series of graded ethanol solutions. The sections were immersed in citrate buffer (0.01 mol/l citric acid, pH 6.0) and heated for two 5 min intervals in a microwave oven for antigen retrieval. Next, the sections were incubated with 0.3% H_2_O_2_ for 15 min to block potential endogenous peroxidase activity. Subsequent to the sections being rinsed in tap water and washed with phosphate-buffered saline (PBS), the sections were incubated with 20% normal goat serum for 30 min at room temperature, then incubated with a monoclonal mouse anti-FLNa antibody (Millipore, Bedford, MA, USA; 1:100) in PBS for 18 h at 4°C. The next day, the sections were washed with PBS three times, then incubated with a goat anti-mouse polymer secondary antibody (Zhongshan Goldenbridge Biotechnology Co., Ltd., Beijing, China) for 30 min at room temperature. Next, DAB plus (Zhongshan Goldenbridge Biotechnology Co.) was added to the sections once they had briefly been washed with PBS and incubated for 5 min to visualize the positive signal. Finally, the sections were counterstained with hematoxylin. Normal skin tissue sections served as the positive control, while normal mammary gland and breast benign tumor tissues served as the negative controls. PBS was used as a blank control instead of the first antibody. The stained tissue sections were reviewed and scored under a light microscope: the intensity of staining was scored as zero (no staining); 1+ (weak cytoplasmic staining in <10% of cells); 2+ (moderate cytoplasmic staining in >10% of cells); and 3+ (marked cytoplasmic staining in >10% of the cells). A score of 0 or 1 was considered to indicate a negative result for FLNa expression (low), whereas scores of 2+ or 3+ were considered to show positive (high) FLNa expression.

### RT-PCR

An RT-PCR analysis was performed to detect FLNa mRNA expression in the snap-frozen breast cancer and distant normal breast tissues. Total RNA from these tissues was isolated using TRIzol reagent (Invitrogen, Carlsbad, CA, USA) according to the manufacturer’s instructions. After measuring the quantity and quality using a spectrophotometer, the total RNA was subjected to reverse transcription into cDNA with 1 μg RNA as a template, using an RT kit (Fermentas, Glen Burnie, MD, USA) in a total volume of 20 μl. PCR amplification was then performed using primers derived from the human FLNa sequence (5′-AGCCTCCACGAGACATCATC-3′ and 5′-CCAGTGTGT ACTCCCCCTTG-3′). The results were normalized to GAPDH, which was used as an internal control. The primer sequences of GAPDH were 5′-GGGTGATGCTGGTGCTGA GTATGT-3′ and 5′-AAGAATGGGAGTTGCTGTTGA AGTC-3′. A semi-quantitative determination of the FLNa mRNA levels was achieved following 30 cycles of PCR amplification. The PCR products were then analyzed using 1% ethidium bromide agarose gel electrophoresis in comparison with the DNA molecular weight marker (Takara, Dalian, China). Finally, a quantitative analysis of the PCR target bands was performed using Image J software (NIH, Bethesda, MD, USA).

### Statistical analysis

SPSS software version 13.0 for Windows (SPSS Inc., Chicago, IL, USA) was used for the statistical analyses. Two-sample t-tests or Mann-Whitney U tests, with respect to data distribution in the measured data, were performed for comparisons between two groups. χ^2^ tests were used for enumeration data. P<0.05 was considered to indicate a statistically significant difference.

## Results

### Overexpression of FLNa protein in breast cancer tissues

**Figure 1:**
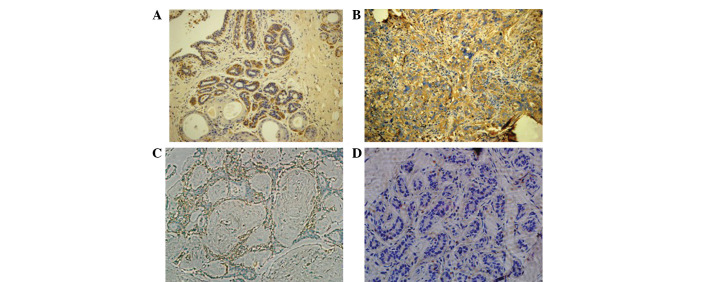
Differential expression of FLNa protein in breast cancer tissue specimens. Immunohistochemical analysis of FLNa protein expression in (A) non-invasive and (B) invasive breast cancer tissues and (C) benign breast tumor and (D) distant normal mammary gland tissues. All images are at ×200 magnification. FLNa, filamin A.

**Figure 2:**
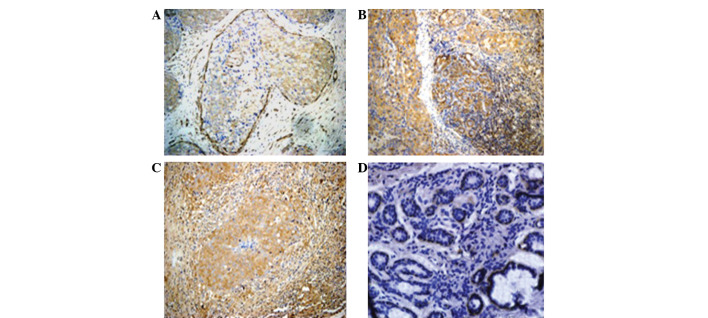
Expression of FLNa protein in breast cancer tissues. Immunohistochemical staining of FLNa protein in (A) Stage I, (B) II and (C) III breast cancer tissues compared with (D) normal breast tissue. All images are at ×200 magnification. FLNa, filamin A.

First, FLNa protein expression was assessed in the breast cancer and distant normal and benign breast tumor tissue specimens (Fig. 1). The data showed that the FLNa protein was mainly detected in the cytoplasm of the breast cancer cells, mostly at the edge of the cells and in the basal cells or intercellular substance (Fig. 1 and 2).

The expression of the FLNa protein was detected in 63.54% of the breast cancer tissues, whereas the immunoreactivity of the FLNa antibody was extremely low in certain distant normal tissues. FLNa was undetectable in the most distant normal breast tissues and benign tumor sections (Fig. 2). Furthermore, in the breast cancer samples, the positive rate of FLNa staining was increased according to the tumor stage, with 36, 66.7 and 84.6% in stage I, II and III breast cancer tissues, respectively (P<0.05).

### Association of FLNa protein expression with clinicopathological features of patients with breast cancer

Next, the associations between FLNa protein expression and the clinicopathological features of the patients with breast cancer were investigated. It was observed that the expression of the FLNa protein was associated with TNM stage, lymph node metastasis, vascular or neural invasion of the tumors, menstruation state and other risk stratifications (P<0.05). However, the expression of the FLNa protein was not associated with any other clinicopathological features, including age, tumor size, localization, histological type and the status of the estrogen and progesterone receptors or the Her2/neu protein (P>0.05; Table I).

### Differential expression of FLNa mRNA in breast cancer and distant non-tumor breast tissues

**Figure 3:**
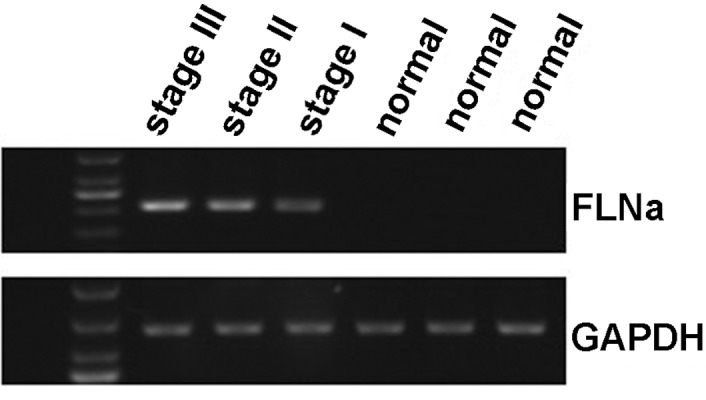
Representative semi-quantitative reverse transcription polymerase chain reaction (RT-PCR) data on FLNa mRNA levels in breast cancer (stages I, II and III) vs. the distant non-tumor tissues. Detection of GAPDH mRNA served as an internal control. FLNa, filamin A.

To investigate whether FLNa expression was regulated at the transcriptional level, a semi-quantitative RT-PCR analysis of FLNa mRNA expression was performed on 30 cases of breast cancer and distant normal tissues. After normalizing to the GAPDH mRNA levels, the expression level of the FLNa mRNA was 0.634±0.53 in the breast cancer tissues and 0.06±0.01 in the distant non-tumor breast tissues (P<0.05; Fig. 3).

## Discussion

In the present study, the differential expression of FLNa mRNA and protein was analyzed in breast cancer tissue specimens in order to provide *ex vivo* data on the potential role of the FLNa protein in breast cancer. It was observed that the FLNa protein was overexpressed in the breast cancer tissues compared with the distant normal mammary gland and benign breast tissues, and that this overexpression was associated with advanced stages, lymph node metastasis and vascular or neural invasion of breast cancer. Semi-quantitative RT-PCR data showed the transcriptional regulation of FLNa overexpression in breast cancer. The present data indicate that FLNa contributes to breast cancer development and progression.

In the present study, FLNa expression was demonstrated in the cytoplasm of the breast cancer cells, mainly at the edge of the cells and in the basal cells or intercellular substance. Until now, it was difficult to determine the association between the function and the localization of FLNa. However, we provide clinical evidence demonstrating that FLNa is frequently overexpressed in breast cancer specimens.

Various other studies have demonstrated an association between FLNa overexpression and tumor metastasis in several types of cancer. For example, the expression of FLNa protein was higher in hepatocellular carcinoma HCCLM9 cells with high metastatic potential compared with hepatocellular carcinoma MHCC97L cells with low metastatic potential (10). The overexpression of FLNa has been associated with the invasion and metastasis of breast cancer (15). Furthermore, FLNa has been shown to affect the invasion and metastatic capacity of lung tumor cells (11). FLNa was reported to be essential for the locomotion of human melanoma cells, and the suppression of FLNa expression inhibited melanoma cell migration and induced apoptosis (12,16). The present study supported these published data. However, two further studies showed contradicting data. The first study by Zhong *et al* reported that the knockdown of cyclinD1 expression suppressed breast cancer cell invasion, which was associated with the downregulation of FLNa protein phosphorylation (13). The second study by Xu *et al* showed that FLNa suppressed the migration and invasion capacity of breast cancer (14). The present *ex vivo* data supported the hypothesis that FLNa expression is associated with breast cancer development and progression. Structurally and molecularly, the FLNa protein functions to crosslink actin filaments into orthogonal networks and serves as the scaffold in various signaling networks (4,5), which in turn has a role in regulating cell shape, adhesion and motility. During tumorigenesis, FLNa may regulate tumor cell invasion and metastasis. Cancer metastasis is, however, a major obstacle for cancer therapy and targeting it may effectively control advanced or aggressive tumors. In addition, the development of novel biomarkers to predict tumor progression, such as metastasis, may also improve cancer survival and reduce fatalities. These biomarkers should distinguish cancers with high metastatic potential from cancers with less metastatic potential, thus optimizing individualized therapeutic planning. However, whether the detection of FLNa protein expression is likely to service as such a biomarker requires further study and verification. Indeed, an additional cohort of tissue samples is likely to aid in the confirmation of the current data.

Previous studies have shown that FLNa expression is positively associated with VEGF, an angiogenesis regulator, in lung cancer (11). FLNa is an important regulatory molecule in the TGF-β signal transduction pathways (17) and is able to bind to SMAD2 to regulate actin polymerization reconciliation and interaction with myosin via β_1_-integrin and RhoA GTPase. Kim *et al*(18) reported that FLNa and β_1_-integrin interacted together to mediate lung cancer (A549) cell proliferation and prevent apoptosis. In addition, FLNa was reported to interact with CEACAM1 (19), P311 (20) and FilGAP (21) to promote tumor cell migration. Ravid *et al*(22) showed that caveolin-1 expression in breast cancer MCF-7 cells upregulated FLNa phosphorylation to induce MCF-7 cell migration through the PI3/AKT pathway. The FLNa protein also interacted with the GTP-binding protein R-Ras to promote metastasis of melanoma cells (23). Together, these findings and the results of the present study clearly support the hypothesis that FLNa is involved in tumor invasion and metastasis. However, it has also been reported that FLNa is able to inhibit MMP-9 expression through Ras/MAPK/ERK signaling to affect tumor cell invasion (24). Thus, future studies should further investigate the role of FLNa in human carcinogenesis and cancer progression.

## Figures and Tables

**Table I:** Association of FLNa protein expression with the clinicopathological characteristics of patients with breast cancer.

Characteristic	n	+, n	−, n	χ^2^	P-value
Age (years)					
≤35	5	4	1	3.154	0.207
35–55	68	45	23		
≥55	23	11	22		
Tumor size (cm)					
≤2	44	23	21	4.76	0.120
2–5	49	34	15		
≥5	3	3	0		
Tumor location					
Left	46	33	13	3.217	0.073
Right	50	27	23		
TNM stage					
I	25	9	16	13.360	0.010
II	45	30	15		
III	26	22	4		
Lymph-node-metastasis					
Yes	53	40	13	8.495	0.040
No	43	20	23		
Vascular/nerve infiltration					
Yes	42	31	11	4.710	0.030
No	52	27	25		
Pathological type					
Invasive	82	52	30	0.201	0.645
Non-invasive	14	8	6		
Histological type					
I	3	2	1	0.998	1.000
II	47	29	18		
III	19	12	7		
Others	27	17	10		
Menstrual state					
Non-pausimenia	60	42	18	4.313	0.038
Pausimenia	35	17	18		
ER					
−	32	21	11	0.316	0.574
+	62	37	25		
PR					
−	43	29	14	1.105	0.293
+	51	29	22		
CerB-b2					
0	60	36	24	0.216	0.975
1+	3	2	1		
2+	11	7	4		
3+	20	13	7		
Risk					
Low	6	2	4	13.426	0.010
Middle	56	29	27		
High	34	29	5		
MBNG					
I	10	6	4	0.725	0.867
II	49	32	17		
III	25	14	11		
Others	12	8	4		

Modified Black’s nuclear grade (MBNG) is a nuclear classification of breast cancer cells. ER, estrogen receptor; PR, progesterone receptor; FLNa, filamin A.
